# Research on the strength prediction equation and model of cement stabilized macadam mixed with recycled construction waste aggregate

**DOI:** 10.1038/s41598-025-94615-9

**Published:** 2025-03-20

**Authors:** Jiangtao Fan, Chen Zhang, Yong Yi, Yu Zhang, Chenfan Bai

**Affiliations:** 1 School of Energy and Architecture, XiHang University, 259 West Second Ring Road, Xi’an, 710077 Shaanxi China; 2 School of Civil and Architectural Engineering, Yangtze Normal University, No. 16, Juxian Avenue, Fuling District, Chongqing, 408100 China; 3 Key Laboratory for Special Area Highway Engineering of Ministry of Education, Chang’an University, South 2nd Ring Rd. Middle Section, Xi’an, 710064 Shaanxi China

**Keywords:** Construction waste, Cement-stabilized macadam, Mechanical properties, Strength prediction equation, Strength prediction model, Environmental sciences, Engineering, Materials science

## Abstract

While the issue of construction waste siege is becoming increasingly serious, the road construction industry is also facing the problem of sand and stone materials shortage, and adding construction waste to the road base can effectively help address both issues. In this work, the effects of 0–9.5 and 9.5–37.5 mm recycled construction waste aggregate (RCWA) content on the mechanical properties of cement-stabilized macadam (CSM) mixed with the RCWA were investigated, with the optimal RCWA content that allows for a strength not lower than that of ordinary CSM determined. Here, the mechanical strength growth law of CSM mixed with RCWA was studied, while a prediction equation and a model of the mechanical strength of the mix were proposed and the attendant reliability verified. The results indicated that the optimal ratio of CSM mixed with RCWA is as follows: 0–9.5 mm RCWA: 9.5–37.5 mm RCWA: 19.5–37.5 mm natural aggregate: 9.5–19.5 mm natural aggregate = 45:20:29:6. The correlation coefficient *R*^2^ of the established strength prediction equation was as high as 0.98, and when the cement content and RCWA content are known, the mechanical strength can be predicted. Meanwhile, the correlation coefficient *R*^2^ of the proposed strength prediction model was as high as 0.99, and when the 7-day mechanical strength of CSM mixed with RCWA is known, the model can be used to predict the mechanical strength at any curing age. This was verified using laboratory tests, and it was found that the deviation between the predicted values and the actual values was small.

## Introduction

With the continuous advancement of urbanization, large-scale reconstruction and expansion have led to an increasing amount of construction waste in terms of, for example, concrete blocks, waste bricks, and muck. Due to the large amounts and the difficulty of disposal, an increasing number of cities are facing severe construction waste disposal issues^1,2^. A large amount of the waste not only occupies land resources but also results in serious pollution within the urban environment^3,4^. Given the scarcity of land resources and the severe environmental problems, how to appropriately deal with construction waste has become an issue that requires urgent resolution^5,6^. CSM is a common semi-rigid base material that is widely used in highway bases due to its good road performance^7–9^. Given that the sub-base and base layer of pavements do not have high requirements in terms of material performance, it is highly feasible to separate the concrete blocks and bricks from construction waste and create recycled aggregate through crushing and processing to replace part or all of the natural aggregate used for CSM bases^10–12^.

Various researchers have investigated transforming construction waste into CSM mixtures for use as the road base. Here, the US state of Michigan took the lead in using recycled concrete for pavement construction and researching the properties of recycled concrete, while Seoul National University^13,14^ analyzed and modified recycled construction waste aggregate (RCWA) to study the compressive strength, shear strength, and fracture resistance of mixtures with different aggregate contents. The results demonstrated that all the indicators met the specification requirements, proving that it is feasible to replace natural aggregates with RCWA for road-base materials. Elsewhere, Arulrajah^15^ analyzed the possibility of using CSM mixed with RCWA, recycled bricks, and broken pebbles as the road base, and suggested that the proportion of recycled bricks should be less than 25%. Meanwhile, Limbachiya^16^ studied the effect of recycled coarse aggregate on concrete, with the results indicating that a 30% proportion of aggregate had little effect on the concrete, while as the content was increased, the strength of the concrete decreased accordingly. In fact, adjusting the water–cement ratio of recycled concrete can ensure that recycled concrete has the same durability and engineering characteristics as concrete mixed with natural aggregates. Nataatmadja^17^ used *E*_c_ (modulus of resilience) as a design index to study the influence of construction waste on CSM bases, with the results indicating that the *E*_c_ of the CSM base layer mixed with construction waste was almost unaffected and thus met the road construction requirements. Elsewhere, researchers from Griffith University in Australia^18,19^ selected various waste concrete blocks with different strength grades to process them into recycled aggregates with different particle size ranges, forming CSM according to the specifications and subsequently testing its mechanical strength. The results indicated that various indicators of RCWA produced through a crushing process met the specification requirements, confirming that recycled aggregate can be used as a filler for cement-stabilized base courses. Meanwhile, Wang et al.^20^ proposed that the mix ratio design had a huge impact on the mechanical strength of CSM and thus focused on the mix ratio of CSM mixed with construction waste. It was also found that the content of construction waste has a huge impact on the *E*_c_ of the mixture and a certain impact on the workability of the mixture. New Mexico State University^21^ studied the elastic modulus of cement-stabilized recycled aggregates, with the results indicating that while CSM mixed with RCWA can be used as the road base, the mix ratio is limited since when the content of RCWA is greater than 25%, the dynamic resilience modulus of the base will be affected to a certain extent. Elsewhere, researchers from Delft University of Technology in the Netherlands^22,23^ studied the relationship between the aggregate properties and the mixture gradation as well as the performance of cement-stabilized recycled aggregates. Here, various triaxial tests were carried out using CSM with different RCWA contents and the results indicated that the gradation had a significant impact on the performance, while when more fine aggregates were added, the internal cohesion of the mixture was stronger. Meanwhile, the content of recycled aggregate has a significant influence on the modulus of the mixture, with the modulus increasing with the increase in RCWA content^24–26^. While all the above results have greatly promoted the application of construction waste in road-base construction, most of the research has only focused on finely sorted RCWA, which includes concrete blocks, bricks, clay, or tailings, and has rarely addressed unsorted RCWA, thus significantly hindering the large-scale utilization of construction waste in road-base construction.

The attendant mechanical properties are also important indicators for evaluating semi-rigid base materials^27^. Since the physical and chemical properties of construction waste are different from those of natural aggregates, the former has the characteristics of large crushing value and high-water absorption, which will inevitably have an impact on the mechanical properties of CSM mixed with construction waste^28,29^. All of the above effects are problems that need to be solved before the large-scale application of CSM mixed with RCWA subgrade, however, at present, it is not universally recognized whether the load-bearing capacity of CSM mixed with RCWA subgrade can meet the requirements. The growth pattern of mechanical strength of CSM mixed with RCWA with the age of regeneration under compression splitting loading is not clear.

In view of this, this paper firstly studied the influence of RCWA on the mechanical strength of CSM, and proposed the optimal doping amount of RCWA in CSM with the strength not lower than that of ordinary CSM. Secondly, the growth law of CSM mixed with RCWA with curing time was studied, and the mechanical strength growth equation and prediction model of CSM mixed with RCWA were established and verified to be reliable. It provides an effective way to realize large-scale resource utilization of construction waste, and to solve the shortage of sand and gravel materials in highway construction and the increasingly serious problem of construction waste surrounding the city.

## Materials and methods

### Raw materials

#### Cement

Zhejiang Guang-Yu brand P.O42.5 cement was used in this work and was tested according to “The Methods of Cement and Concrete for Highway Engineering” (JTG E30-2005)^30^, with the technical indicators shown in Table 1.

**Table 1:** Technical indicators of cement.

Index	Soundness (mm)	Setting time (min)		Flexural strength (MPa)		Compressive strength (MPa)			
		Initial time	Final time	3d	28d		3d	28d	
Test results	1.0	152	312	4.2	5.2		27.6	49.1	

#### Aggregate

The aggregate used was limestone produced in Wuyi County, Jinhua City, Zhejiang Province. The particle size of the aggregate is divided into four grades: 19–37.5, 9.5–19, 4.75–9.5, and 0–4.75 mm and was tested according to “Test Methods of Aggregate for Highway Engineering” (JTG E42-2005)^31^, with the technical indicators shown in Table 2.

**Table 2:** Technical indicators of aggregate.

Aggregate size (mm)	Apparent density (g/cm^3^)	Crushed value (%)	Flakiness content (%)	Water absorption (%)
19–37.5	2.680	—	5.6	0.6
9.5–19	2.657	17.0	8.6	1.1
4.75–9.5	2.630	—	7.2	1.5
0–4.75	2.593	—	—	—

#### Gradation

**Fig. 1:**
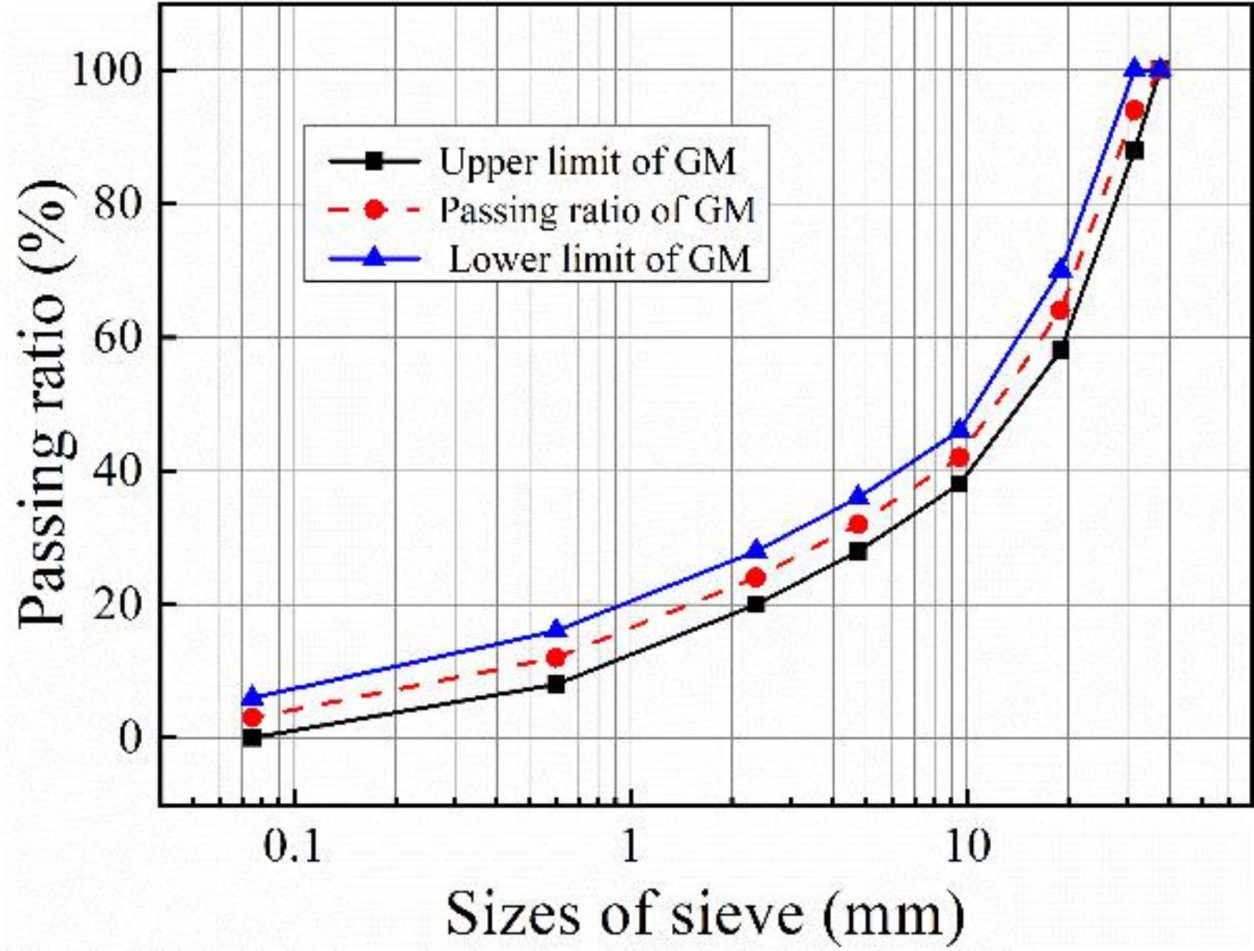
Skeleton-dense grading curve.

Median value of skeleton-dense gradation was used. Table 3 lists the gradation parameters, and Fig. 1 shows the gradation curve, the GM in the figure represents the range of dense skeleton gradation.

**Table 3:** Skeleton-dense gradation.

Grading type	Sieve holes (mm) through mass percentage (%)							
	37.5	31.5	19	9.5	4.75	2.36	0.6	0.075
Skeleton-dense gradation	100.0	94	66	41	32	24	14	4

### Forming method

Various cylinder specimens with a height of 150 mm and a diameter of 150 mm were made for *R*_c_ and *R*_i_ testing according to the static pressure method outlined in JTG E51-2009^32^, and the number of parallel specimens prepared for each set of tests was six.

### Test methods

####  Unconfined compressive strength

Working in accordance with the relevant specifications^32^, after the specimens were cured for 7, 14, 28, 60, and 90 days, the unconfined compressive strength (*R*_c_) tests were performed using a universal testing machine (UTM), with the results calculated according to Eq. (1):

1 $$R_{C} = \frac{P}{A}$$

where *P* is the maximum pressure at the time of specimen failure (*N*), and *A* is the cross-sectional area of the specimen (mm^2^).

#### Splitting strength

Working in accordance with the relevant specifications^32^, after the cylinder specimens were cured for 7, 14, 28, 60, and 90 days, the splitting strength (*R*_i_) tests were performed using the UTM, with the results calculated according to Eq. (2).

2 $$\frac{{R_{T} }}{{R_{a} }} = \frac{{A \cdot \ln ^{B} \left( {T + 1} \right) + C}}{{A \cdot \ln ^{B} \left( {a + 1} \right) + C}}\,\,\,T \le 180d$$

where *d* is the diameter of the specimen (mm), *h* is the height of the specimen following immersion (mm), *P* is the maximum pressure at the time of specimen failure (*N*), *a* is the width of the bead, and *α* is the central angle corresponding to the half bead (º).

## Optimal RCWA content

### RCWA of 0–9.5 mm

#### Aggregate ratio

According to various surveys, more than 80% of the construction waste aggregates processed by factories has grades of 0–9.5 and 9.5–37.5 mm^33^. Therefore, to ensure that the study is in line with the actual situation, we began with the above two grades to study the optimal content.

First, 0–9.5 mm RCWA was mixed with CSM at proportions of 0%, 20%, 30%, 35%, 40%, 45%, 50%, 55%, and 60%, with a cement content of 4% in all cases. Table 4 shows the formulation ratio of the nine different mixtures.

**Table 4:** Aggregate ratio for different proportions of 0–9.5 mm RCWA.

0–9.5 mm RCWA mass percentage (%)	Mass percentage of natural aggregate (mm) under the following particle sizes (%)			
	19–37.5	9.5–19	4.75–9.5	0–4.75
0	40	20	10	30
20	40	20	10	10
30	40	20	0	10
35	40	20	0	5
40	40	20	0	0
45	37	18	0	0
50	37	13	0	0
55	33	12	0	0
60	30	10	0	0

#### Compaction test results

The optimal water content and maximum dry density of the CSM mixed with different proportions of 0–9.5 mm RCWA are shown in Table 5. As Table 5 shows, as the mass percentage of RCWA was increased, the maximum dry density of the CSM gradually decreased, while the optimal water content increased, with the former varying from 1.93 to 2.17 g/cm^3^ and the latter from 5.6 to 7.5%. This was because 0–9.5 mm RCWA has a high-water absorption rate, with the crushed cement mortar particles having a particularly strong ability to absorb water. At the same time, due to the adsorption of water by the abundant pores and cracks on the surface of the RCWA, the optimal water content of the specimens increased. Meanwhile, the decrease in maximum dry density was mainly related to the low density of RCWA. Clearly, the water demand for the mixture to reach maximum compactness is positively correlated with the amount of construction waste.

**Table 5:** Optimal water content and maximum dry density of the mixture with different proportions of RCWA.

Item	Compaction test results of different 0–9.5 mm RCWA mass percentages (%)								
	0	20	30	35	40	45	50	55	60
Optimal water content (%)	5.6	5.9	6.1	6.3	6.5	6.8	7.0	7.3	7.5
Maximum dry density (g/cm^3^)	2.17	2.09	2.07	2.07	2.04	2.02	2.00	1.97	1.93

#### Mechanical properties

**Fig. 2:**
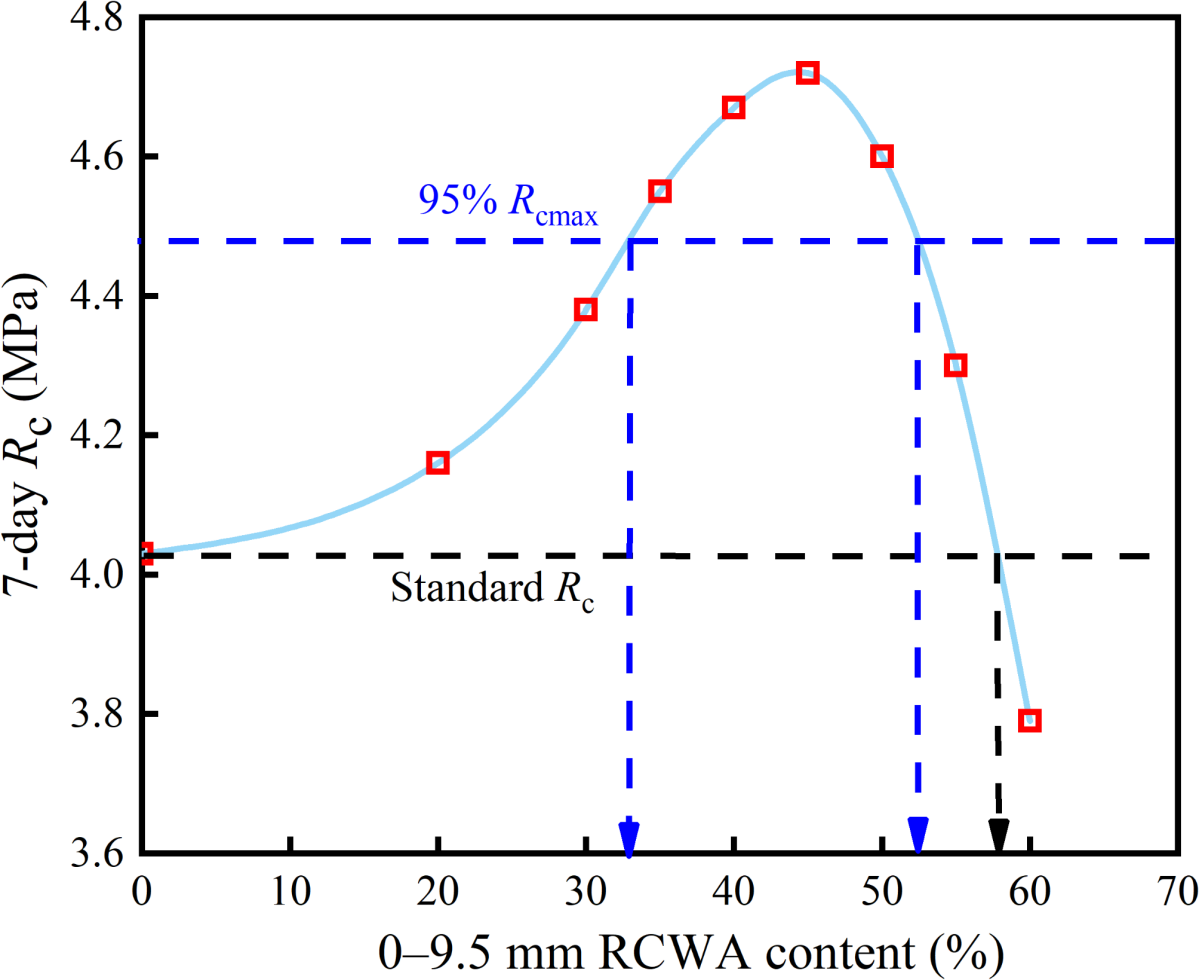
Relationship between 0–9.5 mm RCWA content and *R*_c_

**Fig. 3:**
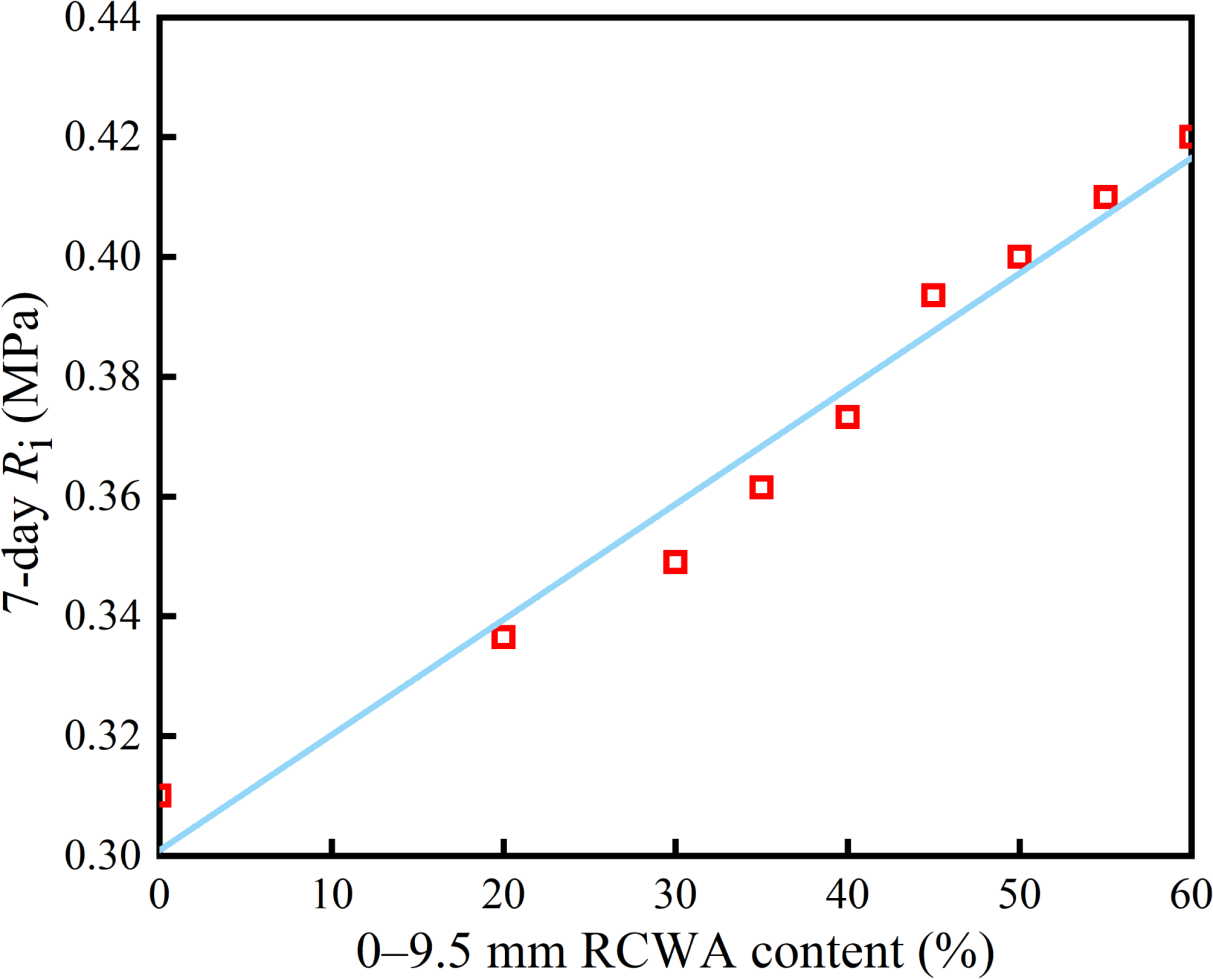
Relationship between 0–9.5 mm RCWA content and *R*_i_.

Figures 2 and 3 show the *R*_c_ and *R*_i_ of the CSM with different contents of 0–9.5 mm RCWA. As Fig. 2 shows, with the increase in RCWA content, the *R*_c_ of the CSM initially increased and then decreased. When the mixing amount was 45%, the *R*_c_ reached the peak value, which was 1.17 times that for no construction waste, while when the mixing amount was 58%, the *R*_c_ was the same as without construction waste. Based on the 95% peak strength, the recommended mixing range is 33–52%, at which point, the *R*_c_ was 1.11 times that without construction waste. This is because CSM is a granular material, and its strength is not only related to the material itself but also to the cementing force of the cement and the embedded extrusion force between the skeletons. Therefore, it can be proposed that the damage of the mixture caused by external forces mainly includes three factors: the damage of the cement mortar, the damage of the interface between the aggregate and the cement, and the damage of the aggregate itself. The 0–9.5 mm RCWA had a large specific surface area and contained specific active substances, which could chemically react with the Ca(OH)_2_ in the cement hydration product to form other hydrates with a specific binding force, thereby enhancing the strength^34^.

When the content of 0–9.5 mm RCWA is less than 45%, the additional strength formed by the construction waste active material will increase the *R*_c_ to a certain extent, offsetting part of the strength loss caused by the reduction in the compression force of the skeleton. At this point, the *R*_c_ increases with the increase in RCWA content. Meanwhile, when the RCWA content is greater than 45%, the *R*_c_ will gradually decrease since the large amount of 0–9.5 mm aggregate will interfere with the structure of the coarse aggregate, causing the skeleton structure of the coarse aggregates to form independent suspension structures. At this point, the aggregate–aggregate interface contacts skeleton strength decreases, while the internal friction within the coarse aggregate will be small, resulting in a decrease in the strength of the mixture.

As Fig. 3 shows, with the increase in RCWA content, the *R*_i_ of the CSM increased linearly, which was because the strength of the natural coarse aggregate was much higher than the strength of the contact surface between the aggregate and the CSM, meaning the fracture surface of the splitting failure mainly existed on the contact surface between the coarse aggregate and the cement hydration product, which confirmed that the *R*_i_ and the adhesion of the contact interface are directly related. Compared with the CSM without construction waste, the hydrate formed by the reaction of the active substance in the construction waste exhibited a level of cohesive force, which improved the interfacial cohesion and resulted in the *R*_i_ gradually increasing with the increase in RCWA content.

### RCWA of 9.5–37.5 mm

#### Aggregate ratio

Following the assessment of the influence of 9.5–37.5 mm RCWA on the performance of CSM, it was determined that the optimal amount of 0–9.5 mm RCWA is 45%. Following this, 9.5–37.5 mm RCWA was added as coarse aggregate in proportions of 5, 10, 15, 20, 25, and 30%, with the cement content 4% throughout.

The 9.5–37.5 mm RCWA was screened, and the natural aggregates of each grade were adjusted according to the screening results to determine the optimal mixing proportion of six different mixtures. The screening results are shown in Table 6, while the aggregate ratios are shown in Table 7.

**Table 6:** RCWA (9.5–3.7.5 mm) screening results.

Sieve aperture (mm)	Mass percentage (%) passing through the sieve aperture (mm)				
	37.5	31.5	19	9.5	4.75
Passing rate (%)	100	97.3	58.8	15.3	0

**Table 7:** Aggregate ratio with different proportions of 9.5–37.5 mm RCWA.

0–9.5 mm RCWA mass percentage (%)	9.5–37.5 mm RCWA mass percentage (%)	Following particle size (mm) natural aggregate mass percentage (%)			
		19-37.5	9.5–19	4.75–9.5	0-4.75
45	0	37	18	0	0
	5	35	15	0	0
	10	33	12	0	0
	15	31	9	0	0
	20	29	6	0	0
	25	27	3	0	0
	30	25	0	0	0

#### Compaction test results

The optimal water contents and maximum dry densities of the CSM mixed with different proportions of 9.5–37.5 mm RCWA are shown in Table 8. As Table 8 shows, the optimal water content of RCWA showed a gradual increase with the increase of 9.5–37.5 mm recycled coarse aggregate dosage. This is mainly due to the fact that the surface of 9.5–37.5 mm aggregate is usually rougher and more angular, and the internal friction resistance between aggregates increases with the increase of its dosage. In order to make the cement paste can better wrap the aggregate particles, reduce the internal friction resistance, so that the aggregate particles can slide and achieve a closer arrangement, it needs more water to play a lubricating role, so the optimal water content gradually increased. And with the increase of 9.5–37.5 mm recycled coarse aggregate mixing, the maximum dry density of RCWA shows a gradual decrease. This is mainly due to the fact that the density of construction waste coarse aggregate is usually smaller than that of ordinary crushed stone, and its materials may include concrete blocks, bricks, etc., which have different densities and some of them are relatively low. When the dosage of construction waste coarse aggregate increases, the dry density of the whole mixture decreases under the same volume due to its own smaller density.

**Table 8:** Optimal water content and maximum dry density of the mixtures with different proportions of 9.5–37.5 mm RCWA.

Item	Compaction test results of different 9.5–37.5 mm RCWA mass percentages (%)					
	5	10	15	20	25	30
Optimal water content (%)	7.0	7.2	7.4	7.7	7.8	8.0
Maximum dry density (g/cm^3^)	1.97	1.95	1.93	1.92	1.90	1.89

#### Mechanical properties

**Fig. 4:**
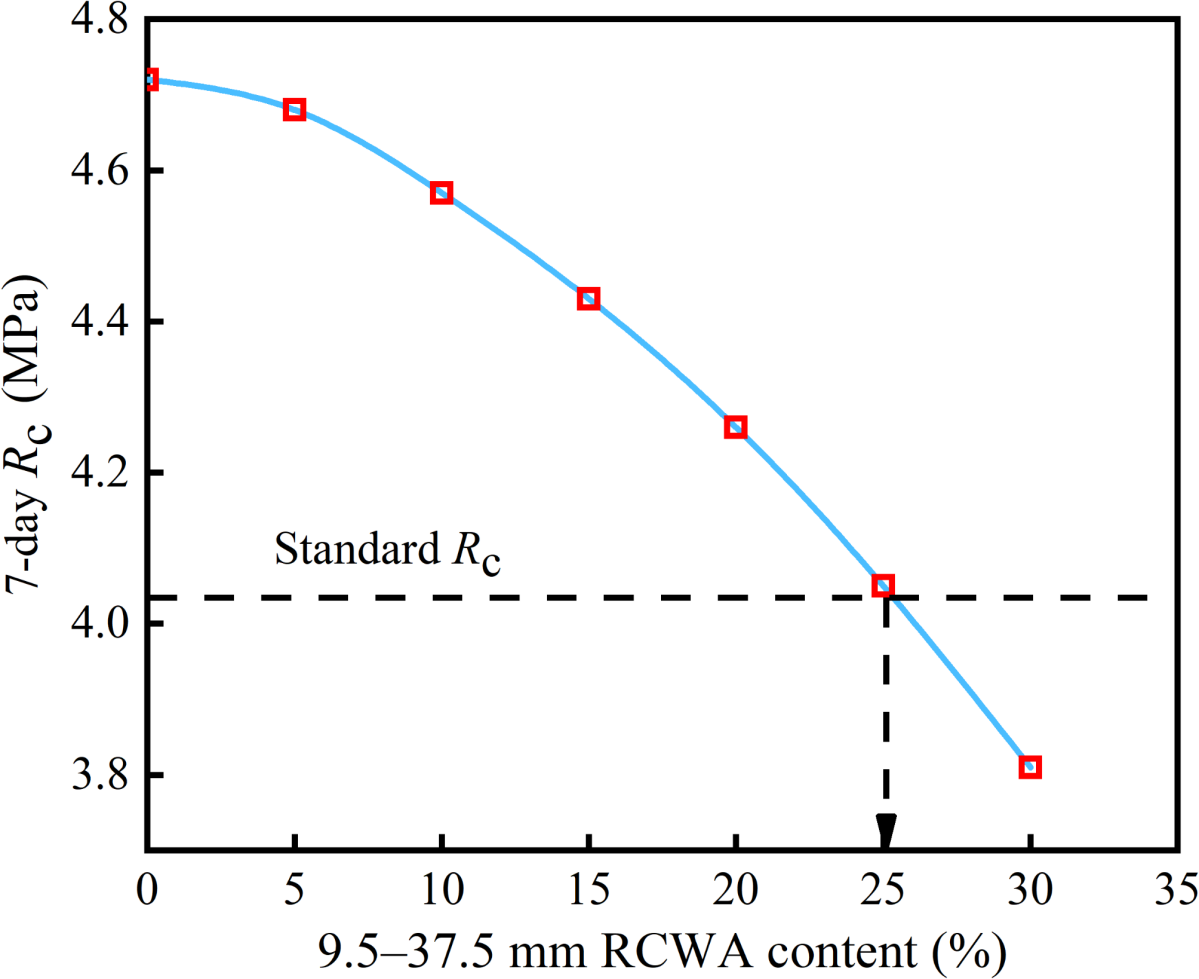
Relationship between 9.5–37.5 mm RCWA content and *R*_c_.

**Fig. 5:**
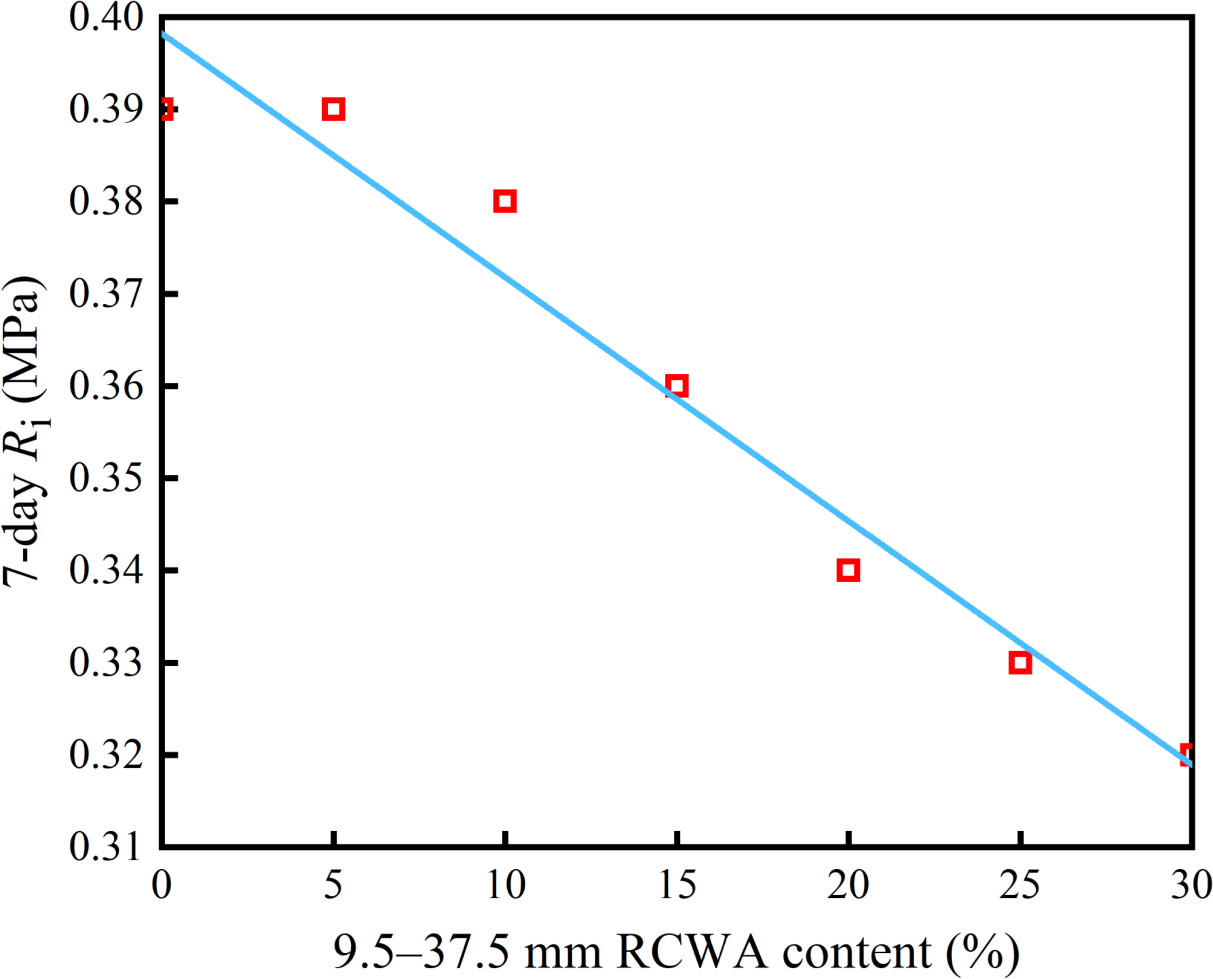
Relationship between 9.5–37.5 mm RCWA content and *R*_i_.

Figures 4 and 5 show the *R*_c_ and *R*_i_ of the CSM with different contents of 9.5–37.5 mm RCWA. As Fig. 4 shows, when the content of 0–9.5 mm RCWA was 45%, the *R*_c_ of the CSM gradually decreased with the increase in the content of 9.5–37.5 mm RCWA. When the content of 9.5–37.5 mm RCWA was around 25%, the strength of the CSM dropped to the strength requirement specified in the code, with the total content of construction waste around 70% at this point. The main reason for the decrease in strength relates to the gradual replacement of natural coarse aggregate with construction waste, that is, the natural aggregate of the mixture skeleton structure transforms part of the recycled construction waste coarse aggregate. However, here, the density of the construction waste coarse aggregate was low, and there existed various voids and cracks inside, while the crushing value was high, resulting in a decrease in the strength of the mixture skeleton, meaning the overall *R*_c_ gradually decreased.

As Fig. 5 shows, when the 0–9.5 mm RCWA content was 45%, with the increase of 9.5–37.5 mm RCWA content, the *R*_i_ of the CSM exhibited a linear decrease trend. This was due to the low strength of the construction waste and the existence of various cracks and voids. During the molding process of the specimen, the coarse aggregate was broken, causing a fracture to develop along the surface of the 9.5–37.5 mm RCWA during the splitting test, resulting in a decrease in the overall *R*_i_.

In summary, when the 0–9.5 mm RCWA content is 45% and the 9.5–37.5 mm RCWA content is 25%, the RCWA can be maximized to meet the specification requirements. However, given that only the 7-day mechanical strength was considered, the test data is slightly insufficient. To ensure that the strength of the CSM with a large amount of construction waste meets the requirements, the mass percentage of the mixture at the optimal content was determined to be as follows: 0–9.5 mm RCWA: 9.5–37.5 mm RCWA: 19.5–37.5 mm natural aggregate: 9.5–19.5 mm natural aggregate = 45:20:29:6.

Meanwhile, to confirm the strength and to ensure that the CSM with a large amount of construction waste is applicable for actual production and use, six mixtures with different aggregate ratios were selected for subsequent tests, as shown in Table 9. Here, *X* and *C* denote the 0–9.5 mm and 9.5–37.5 mm RCWA, respectively, with the numbers indicating the content (e.g., X45C0 denotes a 0–9.5 mm RCWA content of 45% and a 9.5–37.5 mm RCWA content of 0).

**Table 9:** Aggregate ratios.

Type	0–9.5 mm RCWA mass percentage (%)	9.5–37.5 mm RCWA mass percentage (%)	Following particle size (mm) natural aggregate mass percentage (%)			
			19–37.5	9.5–19	4.75–9.5	0–4.75
X0C0	0	0	40	20	10	30
X45C0	45	0	37	18	0	0
X58C0	58	0	30	12	0	0
X45C18	45	18	19	18	0	0
X45C20	45	20	29	6	0	0
X45C25	45	25	27	3	0	0

## Results and discussion

### Laboratory test results

#### Unconfined compressive strength

The heavy compaction method was used to determine the maximum dry density and optimal water content of the CSM specimens mixed with RCWA. Following this, the *R*_c_ of the specimens with different aggregate ratios, cement contents, and curing times was tested, as shown in Table 10.

**Table 10:** *R*_c_ of the CSM specimens mixed with RCWA.

Type	Cement content (%)	*R*_c_ (MPa) of CSM mixed with RCWA in the following curing age (day)				
		7	14	28	60	90
X0C0	3.0	3.5	4.0	4.3	4.4	4.5
	4.0	4.0	4.7	5.1	5.2	5.3
	5.0	4.3	5.0	5.5	5.7	5.8
X45C0	3.0	4.2	4.6	4.9	5.1	5.2
	4.0	4.7	5.3	5.7	5.8	5.9
	5.0	5.1	5.7	6.0	6.2	6.3
X58C0	3.0	3.5	4.0	4.3	4.4	4.5
	4.0	4.0	4.7	5.2	5.3	5.4
	5.0	4.3	5.0	5.6	5.7	5.8
X45C18	3.0	3.3	3.9	4.1	4.2	4.4
	4.0	4.0	4.6	4.8	4.9	5.1
	5.0	4.3	5.0	5.3	5.4	5.6
X45C20	3.0	3.7	4.2	4.4	4.5	4.6
	4.0	4.3	4.8	5.2	5.4	5.4
	5.0	4.5	5.2	5.6	5.8	5.9
X45C25	3.0	3.5	4.0	4.2	4.4	4.5
	4.0	4.1	4.7	5.0	5.2	5.2
	5.0	4.4	5.0	5.5	5.6	5.8

#### Splitting strength

The heavy compaction method was used to determine the maximum dry density and optimal water content of the CSM specimens mixed with RCWA. Following this, the *R*_i_ of the specimens with different aggregate ratios, cement contents, and curing times was tested, as shown in Table 11.

**Table 11:** *R*_i_ of the CSM specimens mixed with RCWA.

Type	Cement content (%)	*R*_i_ (MPa) of CSM mixed with RCWA in the following curing age (d)				
		7	14	28	60	90
X0C0	3.0	0.27	0.35	0.42	0.47	0.50
	4.0	0.32	0.41	0.47	0.51	0.54
	5.0	0.36	0.45	0.50	0.54	0.56
X45C0	3.0	0.34	0.43	0.50	0.55	0.58
	4.0	0.39	0.48	0.54	0.58	0.61
	5.0	0.42	0.50	0.57	0.61	0.63
X58C0	3.0	0.37	0.47	0.55	0.60	0.64
	4.0	0.41	0.52	0.58	0.63	0.67
	5.0	0.44	0.55	0.60	0.64	0.69
X45C18	3.0	0.31	0.41	0.49	0.55	0.56
	4.0	0.35	0.45	0.52	0.57	0.60
	5.0	0.38	0.47	0.53	0.58	0.62
X45C20	3.0	0.31	0.42	0.48	0.53	0.55
	4.0	0.37	0.46	0.51	0.56	0.59
	5.0	0.40	0.48	0.54	0.58	0.61
X45C25	3.0	0.31	0.41	0.49	0.55	0.56
	4.0	0.35	0.45	0.52	0.57	0.60
	5.0	0.38	0.47	0.53	0.58	0.62

Based on the *R*_c_ and *R*_i_ test results, the following conclusions can be drawn:

(1) The cement content had a significant impact on the mechanical strength of the specimens. Under the same aggregate ratio type and curing age, the *R*_c_ and *R*_i_ of the CSM mixed with RCWA increased with the increase in cement content.

(2) The curing age also had a significant influence on the mechanical strength of the specimens. When the aggregate ratio type and cement content are the same, the *R*_c_ and *R*_i_ of CSM mixed with RCWA will increase with the increase in curing age^35,36^ .

(3) Consistent with the previous analysis, the *R*_c_ and *R*_i_ of the CSM mixed with RCWA varied widely with different RCWA contents. Here, the X45C0 specimen had the highest *R*_c_ and X58C0 the highest *R*_i_.

### Mechanical strength growth law

**Fig. 6:**
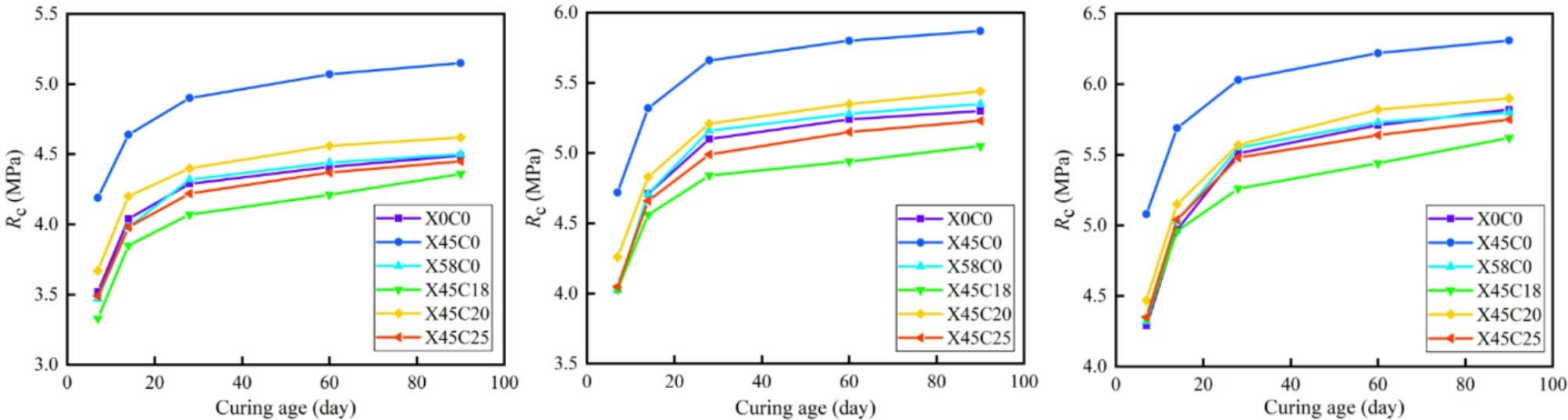
Relationship between *R*_c_ and curing age. (**a**) *P*_s_ = 3% (**b**) *P*_s_ = 4% (**c**) *P*_s_ = 5%.

**Fig. 7:**
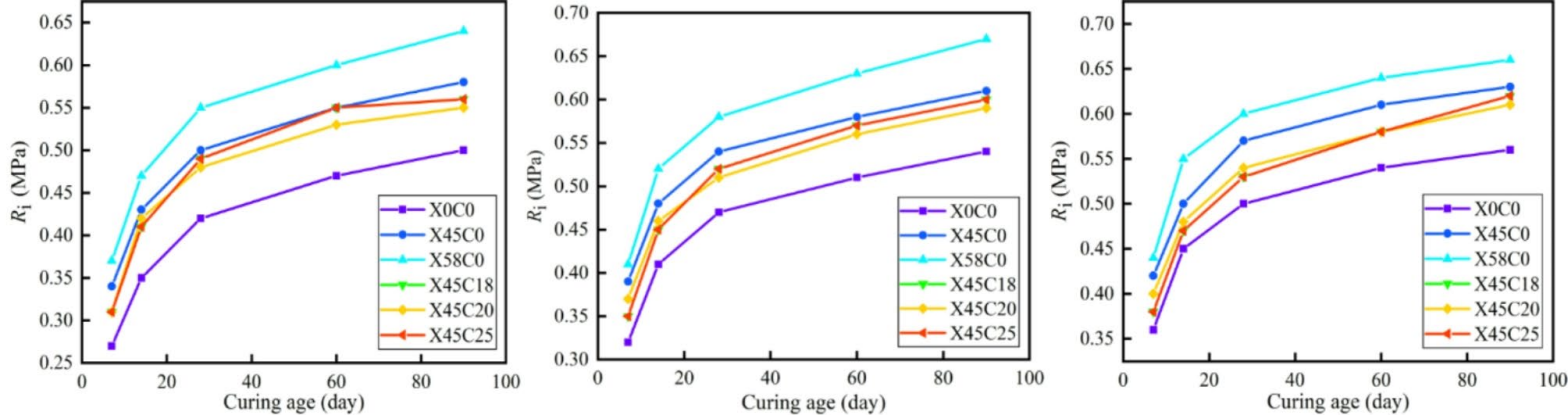
Relationship between *R*_i_ and curing age. (**a**) *P*_s_ = 3% (**b**) *P*_s_ = 4% (**c**) *P*_s_ = 5%.

Based on the data presented in Tables 10 and 11, the relationship between the mechanical strength of the CSM mixed with RCWA and the curing age was assessed, as shown in Figs. 6 and 7. As shown in Figs. 6 and 7, under different recycled aggregate ratios and cement contents, the growth pattern of mechanical strength with curing age of CSM doped with construction waste is basically the same. The mechanical strength curves increased significantly before 28 days and then subsequently flattened. After 90 days of curing, each curve exhibited a horizontal asymptote. The mechanical strength increased with the increase in curing age and was infinitely close to the horizontal asymptote, meaning the horizontal asymptote is the ultimate mechanical strength (*R*_∞_), which is consistent with the research findings of Zhang et al.^37^.

### Strength growth mechanism and equation

#### Strength growth mechanism

The initial strength (*R*_0_) of the CSM relates to the strength of the specimen after curing for 0 days under the premise of the maximum dry density and optimal water content. The *R*_*0*_ is mainly composed of the strength of the mixture itself and a small amount of cement hydration. In the initial stage of curing, the cement is gradually hydrated to form cement stone, which rapidly increases the internal cohesive force of the mixture and gradually increases the mechanical strength of the mixture. Over time, the cement is completely hydrated, no new cement stone is formed, and the mechanical strength of the mixture is no longer improved. At this point, the strength of the mixture is the final strength (*R*_∞_). Therefore, the following assumptions can be made:

3 $$T = o,R_{T} = R_{o}$$

4 $$T = \infty ,R_{T} = R_{\infty }$$

5 $$R_{0} < R_{\infty }$$

where *T* is the curing age (d), *R*_T_ is the mechanical strength curing to age *T* (MPa), *R*_0_ is the initial mechanical strength of the CSM (MPa), and *R*_∞_ is the final mechanical strength of the CSM (MPa).

Based on the above analysis of the strength formation mechanism of CSM, the strength growth equation (Eq. 6) could be proposed and established: When

6 $$R_{T} = R_{\infty } - \frac{{R_{\infty } - R_{0} }}{{\alpha T + 1}}$$

where *R*_0_, *R*_∞_, and *α* are the regression coefficients of the strength growth equation.

#### Strength growth equation

Given that Eq. (6) does not exist in the original software, a custom function (Eq. 6) was added and used to fit the data. Tables 12 and 13 present the regression coefficients of *R*_c_ and *R*_i_. Here, it should be noted that for the CSM specimens, *R*_i0_ = 0^38^, while the subscripts *c* and _*0*_ of *R*_c0_ represent the compressive strength and curing age, respectively.

According to Tables 12 and 13, the correlation coefficients (*R*^2^) were all greater than 0.98, indicating that the established strength prediction equation of CSM mixed with RCWA can accurately predict the growth law of the mechanical strength of the mixture at various cement and RCWA contents.

**Table 12:** *R*_c_ growth equation parameters of the CSM mixed with RCWA.

Ratio type	Cement content (%)	α	*R*_c0_	*R*_c∞_	*R* ^2^
X0C0	3.0	0.3	1.49	4.6	0.99
	4.0	0.3	1.47	5.4	0.99
	5.0	0.3	1.44	5.9	0.99
X45C0	3.0	0.3	1.53	5.3	0.99
	4.0	0.3	1.54	6.1	0.99
	5.0	0.3	1.55	6.6	0.99
X58C0	3.0	0.3	1.48	4.6	0.98
	4.0	0.3	1.45	5.5	0.99
	5.0	0.3	1.45	5.9	0.99
X45C18	3.0	0.3	1.46	4.4	0.99
	4.0	0.3	1.51	5.2	0.99
	5.0	0.3	1.49	5.7	0.99
X45C20	3.0	0.3	1.53	4.6	0.98
	4.0	0.3	1.50	5.6	0.99
	5.0	0.3	1.51	6.1	0.99
X45C25	3.0	0.3	1.49	4.5	0.99
	4.0	0.3	1.48	5.4	0.99
	5.0	0.3	1.47	5.9	0.99

**Table 13:** *R*_i_ growth equation parameters of the CSM mixed with RCWA.

Ratio type	Cement content (%)	β	*R*_i∞_	*R* ^2^
X0C0	3.0	0.105	0.55	0.99
	4.0	0.105	0.58	0.99
	5.0	0.105	0.60	0.99
X45C0	3.0	0.105	0.62	0.99
	4.0	0.105	0.65	0.99
	5.0	0.105	0.67	0.99
X58C0	3.0	0.105	0.68	0.99
	4.0	0.105	0.71	0.99
	5.0	0.105	0.72	0.99
X45C18	3.0	0.105	0.62	0.99
	4.0	0.105	0.65	0.99
	5.0	0.105	0.66	0.99
X45C20	3.0	0.105	0.60	0.99
	4.0	0.105	0.62	0.99
	5.0	0.105	0.65	0.99
X45C25	3.0	0.105	0.62	0.99
	4.0	0.105	0.64	0.99
	5.0	0.105	0.66	0.99

#### Strength growth model

**Fig. 8:**
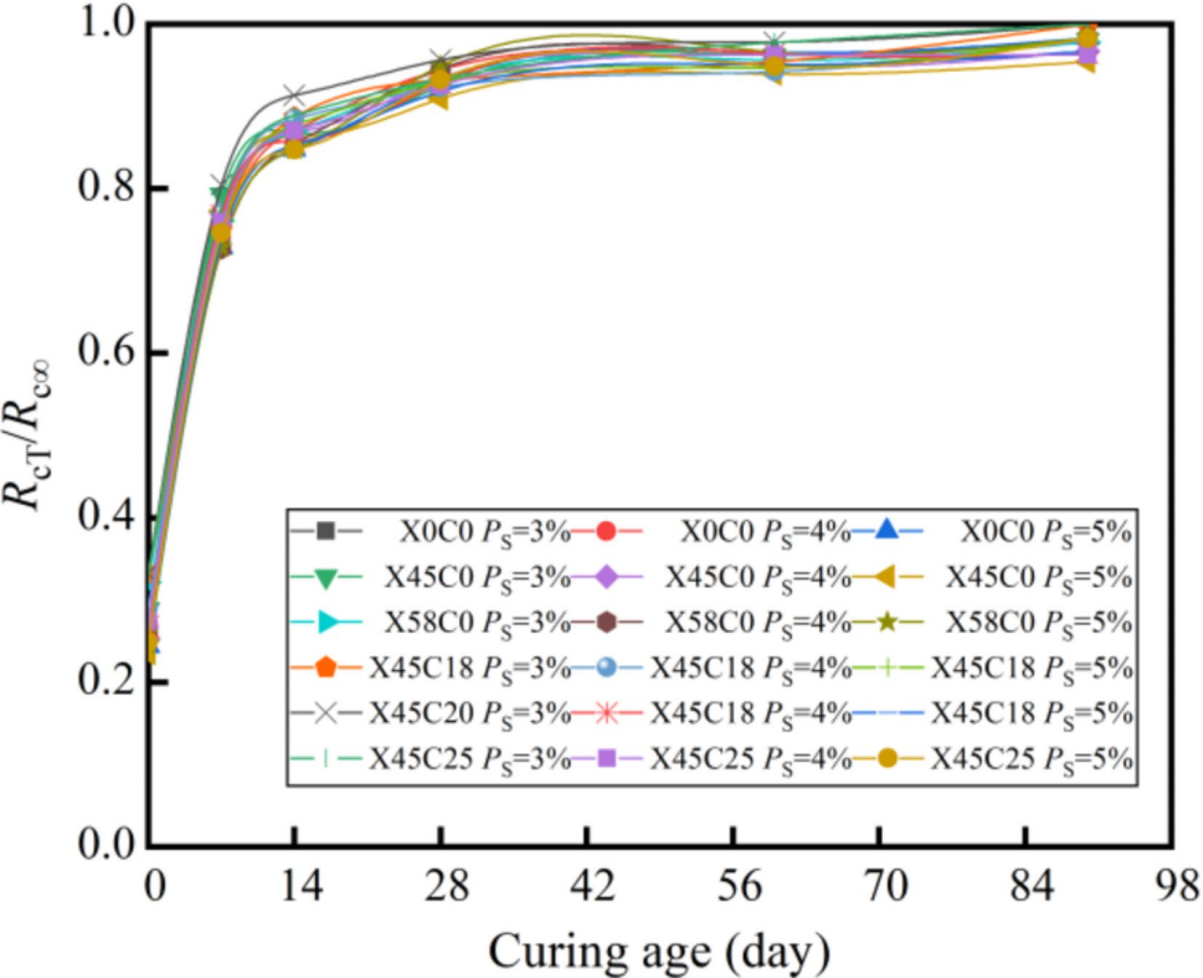
*R*_cT_/*R*_c∞_–*T*.

Figures 8 and 8 show the relationships of *R*_cT_/*R*_c∞_–*T* and *R*_iT_/*R*_i∞_–*T*, where *R*_cT_/*R*_c∞_ represents the compressive strength ratio of the CSM mixed with RCWA at a curing time of *T* to the ultimate compressive strength, while *R*_iT_/*R*_i∞_ represents the splitting strength ratio of the CSM mixed with RCWA at a curing time of *T* to the ultimate splitting strength.

As shown in Figs. 8 and 8, the *R*_T_/*R*_∞_ of the CSM mixed with RCWA at different cement and RCWA contents can be normalized with an increase of the curing age. This means that when the curing age is the same, the RCWA content and cement content have little effect on the *R*_T_/*R*_∞_. Therefore, we could take the average *R*_T_/*R*_∞_ value of the CSM mixed with RCWA under different curing ages and draw the relationship between the average of *R*_T_/*R*_∞_ value and ln(*T* + 1), as shown in Fig. 10.

As Fig. 10 clearly shows, the relationship between *R*_T_/*R*_∞_ and ln(*T* + 1) of the CSM mixed with RCWA conformed to the power function:

7 $$\frac{{R_{T} }}{{R_{\infty } }} = A \cdot \ln ^{B} \left( {T + 1} \right) + C\,\,\,T \le 180d$$

where *A* and *B* are the regression coefficients.

**Fig. 9:**
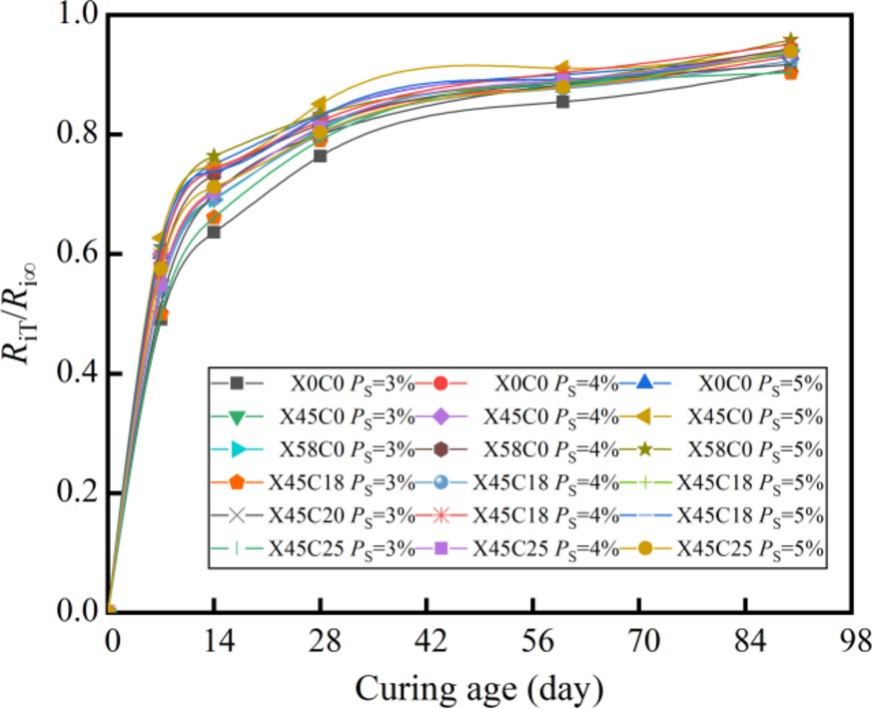
*R*_iT_/*R*_i∞_–*T*.

**Fig. 10:**
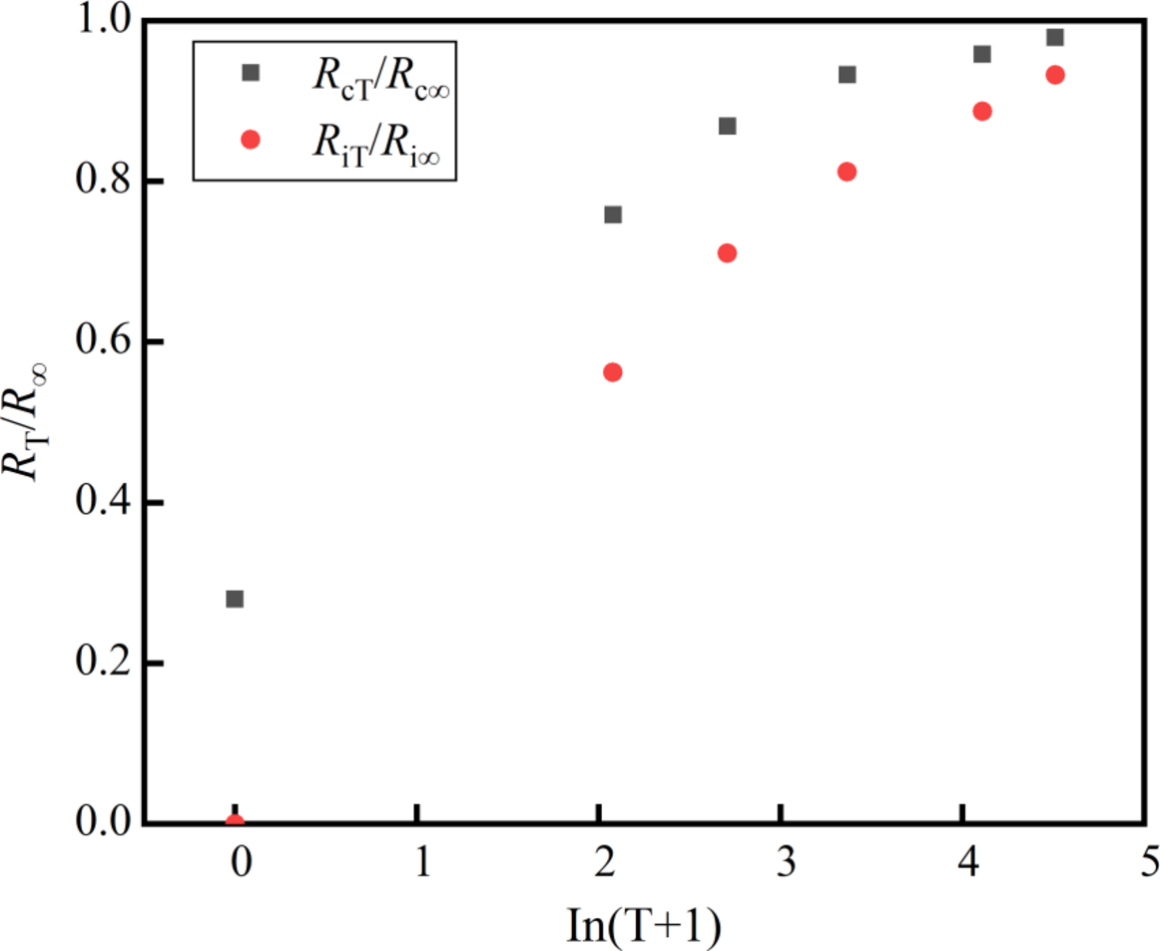
*R*_T_/*R*_∞_–In(*T* + 1).

Following this, Eq. (7) was fitted in the original software, with the results shown in Table 14. Here, *A*_c_, *B*_c_, and *C*_C_ are the regression coefficients of the *R*_c_ equation, and *A*_i_, *B*_i_, and *C*_i_ are the regression coefficients of the *R*_i_ equation.

**Table 14:** Regression coefficients *A*, *B*, and *C.*

Regression coefficients	*R*_cT_/*R*_c∞_–ln(T + 1)					*R*_iT_/*R*_i∞_–ln(T + 1)			
	A_c_	B_c_	C_C_	*R* ^2^		A_i_	B_i_	C_i_	*R* ^2^
Value	0.365	0.447	0.279	0.99		0.376	0.612	0	0.99

According to Table 14, the correlation coefficients, *R*^2^, of the relationship between *R*_T_/*R*_∞_ and ln(*T* + 1) of the CSM mixed with RCWA fitted by the power function were both 0.99, which was sufficient for confirming that Eq. (7) can characterize the growth law of the mechanical strength of CSM mixed with RCWA. In summary, the mechanical strength prediction model of CSM mixed with RCWA was established as follows:

8 $$\frac{{R_{T} }}{{R_{a} }} = \frac{{A \cdot \ln ^{B} \left( {T + 1} \right) + C}}{{A \cdot \ln ^{B} \left( {a + 1} \right) + C}}\,\,\,T \le 180d$$

where *R*_T_ and *R*_a_ are the mechanical strength of CSM mixed with RCWA at a curing age of *T*(d) and *a*(d) (MPa), and *A*, *B*, and *C* are the model coefficients shown in Table 14.

According to Eq. (8), when the *R*_c_ and *R*_i_ of CSM mixed with RCWA at a curing age of 7 days are determined, the *R*_c_ and *R*_i_ of CSM mixed with RCWA at any curing age (*T*>7) can be predicted.

## Case studies

The recycled construction waste produced by Zhejiang Jinhua Zhongtian Urban Construction Green Renewable Resources Co., Ltd. and broken limestone produced by Jinhua were used to verify the reliability of the mechanical strength growth equation and the strength prediction model of the CSM mixed with RCWA proposed in this paper. The *R*_c_ and *R*_i_ of the CSM mixed with RCWA under different cement contents (3.0% and 4.0%) and curing ages (7, 14, 28, and 60 days) were tested in the laboratory, with construction waste ratios of X0C0, X45C0, and X45C20. The measured 7-day *R*_c_ and *R*_i_ of the X0C0, X45C0, and X45C20 CSM mixed with RCWA with a cement content of 3% were 3.5, 4.1, and 3.8 MPa and 0.28, 0.34, and 0.30 MPa, respectively, while the 7-day *R*_c_ and *R*_i_ of the X0C0, X45C0, and X45C20 CSM mixed with RCWA with a cement content of 4% were 4.1, 4.7, and 4.3 MPa and 0.33, 0.39, and 0.37 MPa, respectively.

### Evaluation of the strength growth equation

Based on Eq. (6), the *R*_c_ and *R*_i_ of CSM mixed with RCWA at curing ages of 14, 28, and 60 days were predicted. Here, the parameters listed in Tables 12 and 13 were used for the prediction, with the results shown in Table 15 along with the corresponding indoor measurement values. As Table 15 shows, the maximum error between the measured values and the predicted values of the strength growth equation proposed in this work did not exceed 14%. This confirmed that after determining the cement content and construction waste content, the strength growth equation (Eq. 6) can be used to accurately predict the mechanical strength of CSM mixed with RCWA at any curing age. This is important for guiding the gradation design of CSM mixed with RCWA.

**Table 15:** Measured and predicted values of the strength growth equation.

Mechanical indexes	Type	Cement contents (%)	Mechanical strength (MPa) of CSM corresponding to the following curing time (d)								
			14			28			60		
			Measured values	Predicted values	Error (%)	Measured values	Predicted values	Error (%)	Measured values	Predicted Values	Error (%)
*R*_c_	X0C0	3.0	4.11	4.00	-2.7	4.42	4.27	-3.4	4.39	4.44	1.1
		4.0	4.52	4.64	2.7	5.14	4.98	-3.1	5.16	5.19	0.6
	X45C0	3.0	4.69	4.58	-2.3	4.97	4.90	-1.4	5.18	5.10	-1.5
		4.0	5.35	5.22	-2.4	5.53	5.61	1.4	5.75	5.86	1.9
	X45C20	3.0	4.26	4.01	-5.9	4.37	4.27	-2.3	4.56	4.44	-2.6
		4.0	4.86	4.81	-1.0	5.07	5.16	1.8	5.32	5.38	1.1
*R*_i_	X0C0	3.0	0.34	0.33	-2.9	0.43	0.41	-4.7	0.46	0.47	2.2
		4.0	0.40	0.35	-12.5	0.46	0.43	-6.5	0.52	0.50	-3.8
	X45C0	3.0	0.41	0.37	-9.8	0.50	0.46	-8.0	0.55	0.54	-1.8
		4.0	0.45	0.39	-13.3	0.55	0.49	-10.9	0.57	0.56	-1.8
	X45C20	3.0	0.40	0.36	-10.0	0.46	0.45	-2.2	0.52	0.52	0
		4.0	0.43	0.37	-14.0	0.52	0.46	-11.5	0.56	0.54	-3.6

### Evaluation of the strength growth model

Based on Eq. (8), the *R*_c_ and *R*_i_ of the CSM mixed with RCWA at curing ages of 14, 28, and 60 days were predicted. Here, the parameters listed in Table 14 were used for the prediction, with the results shown in Table 16 along with the corresponding indoor measurement values. As Table 16 shows, the maximum error between the measured value and the predicted value of the proposed strength prediction model did not exceed 13%, meaning the prediction model can accurately predict the mechanical strength of CSM mixed with RCWA at any curing age when the 7-day mechanical strength is known. This could effectively reduce the number of tests and greatly reduce the workload. Furthermore, when the supply of raw materials is insufficient, the prediction model can effectively resolve this problem, and, to a certain extent, can allow for avoiding long-term mechanical strength testing, thus presenting extremely high promotion value.

**Table 16:** Measured and predicted values of the strength growth model.

Mechanical indexes	Type	Cement contents (%)	Mechanical strength (MPa) of CSM corresponding to the following curing time (d)								
			14			28			60		
			Measured values	Predicted values	Error (%)	Measured values	Predicted values	Error (%)	Measured values	Predicted Values	Error (%)
*R*_c_	X0C0	3.0	4.11	3.78	-8.0	4.42	4.04	-8.6	4.39	4.30	-2.1
		4.0	4.52	4.43	-2.0	5.14	4.74	-7.8	5.16	5.04	-2.3
	X45C0	3.0	4.69	4.43	-5.5	4.97	4.74	-4.6	5.18	5.04	-2.7
		4.0	5.35	5.08	-5.0	5.53	5.43	-1.8	5.75	5.78	0.5
	X45C20	3.0	4.26	4.11	-3.5	4.37	4.39	0.5	4.56	4.67	2.4
		4.0	4.86	4.65	-4.3	5.07	4.97	-2.0	5.32	5.29	-0.6
*R*_i_	X0C0	3.0	0.34	0.33	-2.9	0.43	0.38	-11.6	0.46	0.42	-8.7
		4.0	0.40	0.39	-2.5	0.46	0.44	-4.3	0.52	0.50	-3.8
	X45C0	3.0	0.41	0.40	-2.4	0.50	0.46	-8.0	0.55	0.52	-5.5
		4.0	0.45	0.46	2.2	0.55	0.52	-5.5	0.57	0.59	3.5
	X45C20	3.0	0.40	0.35	-12.5	0.46	0.40	-13.0	0.52	0.46	-11.5
		4.0	0.43	0.43	0	0.52	0.50	-3.8	0.56	0.56	0

In summary, the established strength prediction equation and strength prediction model can accurately predict the mechanical strength and growth law of CSM mixed with RCWA. However, the existence of various complex conditions, such as cement performance indicators, aggregate segregation, and curing environment, will affect the strength and growth law, meaning the proposed prediction equation and prediction model still have certain limitations. Therefore, in subsequent experiments, the aforementioned complex conditions will be studied, and the established strength prediction equations and prediction models will be improved to enhance their applicability and practical value.

## Conclusions

In this paper, the optimal content of RCWA, a mechanical strength prediction equation, and a mechanical strength prediction model of CSM mixed with RCWA were studied, with the following conclusions drawn.

(1) In terms of the effect of 0–9.5 mm RCWA on the mechanical strength of CSM, the results indicated that the *R*_c_ gradually increased with the increase in RCWA content, with the strength reaching the peak when the content was 45%. When the content was 58%, the strength was the same as that without construction waste. The recommended content range for the 95% strength peak is 33–52%.

(2) In terms of the effect of 9.5–37.5 mm RCWA on the mechanical strength of CSM when the 0–9.5 mm RCWA content was 45%, the results indicated that the *R*_c_ gradually decreased with the increase in 9.5–37.5 mm RCWA content, while the strength when the content was 25% was the same as that without construction waste. The following optimal CSM mixed with RCWA ratio was proposed: 0–9.5 mm RCWA: 9.5–37.5 mm RCWA: 19.5–37.5 mm natural aggregate: 9.5–19.5 mm natural aggregate = 45:20:29:6.

(3) The correlation coefficient *R*^2^ of the proposed strength prediction equation exceeded 0.98, indicating that the mechanical strength of CSM mixed with RCWA can be accurately predicted after the cement content and RCWA content are determined.

(4) The correlation coefficient *R*^2^ of the proposed strength prediction model was as high as 0.99, which indicated that after the 7-day mechanical strength has been determined, the model can accurately predict the mechanical strength of CSM mixed with RCWA under any curing age.

(5) Through actual engineering verification, it was found that the deviation between the mechanical strength of the CSM mixed with RCWA obtained using the strength prediction equation and the prediction model and the experimental measured values was small, which demonstrates that the established strength prediction equation and model have great promotion value.

## Limitations and future work

In this paper, the optimal content of RCWA, a mechanical strength prediction equation, and a mechanical strength prediction model of CSM mixed with RCWA were studied, but there are still some limitations that can be addressed in future research.

(1) This study only focuses on the mechanical property changes of CSM mixed with RCWA in a relatively short period of time, and does not carry out an in-depth study on their long-term durability and stability. In the future, a long-term monitoring mechanism will be established to track the changes in the performance of CSM mixed with RCWA in the actual use process, and to study its durability and stability, so as to provide a scientific basis for the long-term maintenance and management of roads.

(2) This paper does not carry out a comprehensive economic cost analysis and environmental benefit assessment. In practical application, economic cost and environmental impact are important considerations, and the lack of such analysis will affect the practical application value of the research results. The next step will be to carry out a comprehensive economic cost analysis, including the costs of raw material procurement, production and processing, construction and other aspects, to assess its economic advantages compared with the traditional CSM.

## Data Availability

Data availability statement: The data are available from the corresponding author on reasonable request.
